# Supplementary material for: Cytological and Molecular Characterization of a New Ogura Cytoplasmic Male Sterility Restorer of Brassica napus L
Source: Plants (Basel). 2024 Jun 19;13(12):1703. doi: 10.3390/plants13121703 (PMC11207357; doi:10.3390/plants13121703)
Supplement: Supplementary file 1 [file plants-13-01703-s001.zip › plants-3041040-supplementary.pdf]

Supplementary Table S1: The sequences of 79 molecular markers

Sort	Marker Group	Coded Marker	Primer Sequence（5'-3'）	Size in bp
01	I	RMA01	S: GCTTCTACTTCCATACCAATGG<br>A: CAAGCTCTTCGGTATGAAACG	247
02	I	RMA02	S: AAGCTTCAGCTTATCCTTGG<br>A: GTTCGTTGTAGATCGGATCC	198
03	I	RMA03	S: CTTGCTGCAAAGCACTTCTC<br>A: AGCTTCAGACCAAGTCCCAG	233
04	I	RMA04	S: GGATCACGAAACTCCCAAGG<br>A: TCATATCTCCCTCCTTGTCCA	348
05	I	RMA05	S: AAGCTCAGGCTCCTTCACCG<br>A: GGGAAGGAGATCCGGACTCA	581
06	I	RMA06	S: AAGCTTATAGAGTAGCCATTGAG<br>A: TCTAAGATCAGTATATGGACAGC	249
07	I	RMA07	S: CGGACTCTTTAGCTCCGCCA<br>A: CACCTCCTGTCGGCATCTCA	350
08	I	RMA08	S: TATTCTGCTTCATGTGGTGATC<br>A: ACGATTGTTAAGTTGACGAAAG	354
09	I	RMA09	S: TTTTTCAATGCTTCTGTGCAG<br>A: GCACAAAATTACAATCAGCGC	357
10	I	RMA10	S: AAGCTTTGTGTTGCTAATGTAT<br>A: AGTTGAAACGATATAACTTGTGA	208
11	II	RMB01	S: ATTGTCGTTGTCGATGCATC<br>A: AGAAGAAGAAAGTGCCAAGCA	572
12	II	E35M62	S: AAAATTGCGAGGTTCAGGAAT<br>A: CTCCAGCTCCTGTTAGTGACTCTT	215
13	II	RMB02	S: AATTTATGGGGTGTCAATTGA<br>A: TGGCTGATTTGCAACATAAA	301
14	II	RMB03	S: GTTCTGGCTATGTCGAGACCAC<br>A: CCAGAGTTTGGAGGCAGACT	459
15	II	RMB04	S: GAGTTGTGGGTTTGGCCGTC<br>A: ACGCACCAGAACGATCAATC	168
16	II	RMB05	S: ATCAGAGCAAAAGAGTGCGTAG<br>A: CGAAATACCGAAGAACCAAATC	325
17	II	RMB06	S: ACATCGGTCGAAGAAGTTCC<br>A: AATCTTGAGGCAAGCCTGAC	504
18	II	RMB07	S: AGCTTCTATTCAGCCAAAAGG<br>A: GCATTACCGTTGGAAAATTTC	537
19	II	RMB08	S: ACCAAAGACACCATAACGAGG<br>A: CGCACTTTTAGCAGCAGTTC	524
20	II	RMB09	S: CCCACTCTTGTTACCTTCAGC<br>A: GTTCCCACAGCCTACCAGTAC	316

Sort	Marker Group	Coded Marker	Primer Sequence（5'-3'）	Size in bp
21	II	RMB10	S: ATTGGATTTGAATGAGATGG<br>A: TCCATTGATCTCTGCACATC	358
22	II	OPF10	S: AACTTTTTGTGTTTGATTTCTTGC<br>A: ACTCCTTCTAAACAAAACCAAACA	496
23	II	RMB11	S: AAGCTTGTCTCCTACGTACTTC<br>A: TCAGAAAGATATTTCACGTCAC	317
24	II	RMB12	S: TGGACTAAGAAAGGGTCAGGTA<br>A: CGAAGAATCTCTACTCTGTTGT	750
25	III	RMC01	S: AGGAAGTGAGAGGCAGTTGG<br>A: TCCATGGGTGTCCTAGGATC	356
26	III	RMC02	S: TGCGTAACACTTCTTTGCTTC<br>A: TGCAGAACTCAAAGCCATTC	479
27	III	RMC03	S: AAGCTTATTTTCATCCTGCAA<br>A: CATCACCATCATCACAGTAATT	266
28	III	E38M60	S: TCCATAGAAGAAACTCTTTGCAAC<br>A: TCGACACACTTACTAATCTGAGAGTG	116
29	III	RMC04	S: TATTTTGTCCTCGGTTAGATC<br>A: TTCCTTTGTGTTTGGTTAGGG	213
30	III	RMC05	S: TGCGAGTTTAATCCGGACGC<br>A: CCGCGTTATTCTGGTTCAGAGA	500
31	III	RMC06	S: TTCCTCGGCAAGAACAACGC<br>A: GCCGTCTAACAGCAGGTGCA	482
32	III	RMC07	S: CCGTATTTGAAAACGTGGCG<br>A: TCAACCGTGAATTTGGGTCG	466
33	III	RMC08	S: GAGGCGAAAACATAAACAAGG<br>A: ATCGCCAAAACTGTTTCAGG	547
34	III	RMC09	S: TCGGTTTTTCGAGGGTATCA<br>A: TCCGATTTAGAATCGAACCTG	327
35	III	RMC10	S: TCCTGCAGTTTGAAATCCTTG<br>A: AAGTTTCCCCAAACCAACTTC	465
36	III	RMC11	S: AAGCTTAATAGCGACTTCTTC<br>A: TGAAAACCCTAGTCTCTCTCTC	273
37	III	RMC12	S: AATGGATGAACTCGAGACGG<br>A: TGATAACCCCTCGTTTCCTG	347
38	III	RMC13	S: TGTCAGCATTCAGCAGAAGC<br>A: AGGGATTGAAAGCTGGGAAC	382
39	III	RMC14	S: TTGACGGTTACCCAAAATACCG<br>A: TTGATTGCTTCACCCTCACCC	533
40	III	RMC15	S: AAAGCATCCTTTGCAAGGGG<br>A: GAACCAAAAATGAGTGGATGG	711
41	III	RMC16	S: AAATTGTTACAAAGTATGAGAAATG<br>A: TTCAGTAAACATTTTACTCATTCTC	400

Sort	Marker Group	Coded Marker	Primer Sequence（5’-3’）	Size in bp
42	III	RMC17	S: TTTCCACACAAATCGGATTTAA<br>A: TGGCCAATGAAAGTTTACTGAT	554
43	III	RMC18	S: ACCAAACCGAGAACAAAATAGGTG<br>A: GGTTCGAATACTTTGGTTTTTTGG	525
44	III	RMC19	S: TGGAGGTGTCAAAGTGTGGC<br>A: CGCAAGTCACTTTATTTGGC	543
45	III	RMC20	S: GAACCACGACTTTGGGTCTG<br>A: GCTTTGGTTAGAATGTCGGC	463
46	III	RMC21	S: GAGAATATTGGAAGAAAGCGG<br>A: AAGTCGTGGTTCCTTTGAGG	269
47	III	RMC22	S: GCTCTACGAGTGAGGATCAAAG<br>A: CACTTTCGGAATCCAAGCTC	747
48	III	RMC23	S: AGCTTATAGGCTTCTAGACCC<br>A: GTTTCTGTTTCTGCAGGCTC	219
49	III	RMC24	S: AGCTTTAATTCATGTATTTTTACA<br>A: AATTTTTTTGTGATACATTTCAA	363
50	III	OPC2	S: CTGTAACTTTCAACCCAACTCGTAGAA<br>A: TTTTGGGGATTACTCTTCTTAGCTTTC	678
51	III	RMC25	S: AAGCTTGATCAAAGATCACAG<br>A: AACAAACTAATGAGCAACAGG	364
52	III	RMC26	S: CAGACCGTTCAAGTTCATGG<br>A: CAAGTTGCTCGGCATATGAT	201
53	III	RMC27	S: CCTTCTCCAAACCGGTAAAC<br>A: TTTTGAGAAATGACGGATCG	238
54	III	RMC28	S: AGACCAAGAGGAAGCGTAGC<br>A: AAGAAACAACCCAGACTCCG	623
55	III	RMC29	S: CAATGATTTATACTTCGTTTTTGC<br>A: GCAGCGTACGGTATGTCTATCT	198
56	III	RMC30	S: CATTTGGTTTGTCCGTGTGT<br>A: AGGCGACAACCTCTTTCAAC	525
57	III	RMC31	S: CATTTTCTTTAACAACGCGC<br>A: ACGACGGCGACATGTAGTAC	379
58	III	RMC32	S: TCTCTCACACTTTCTCTCAC<br>A: CGCCGAGAATTTCCGCGCC	450
59	III	RMC33	S: CAAATCAATACCATTAAAAGTGG<br>A: TTTTTGATTAATTTCCTTTCACA	275
60	IV	E33M47	S: AATAGAGGGAGAGGATGAAAGAAC<br>A: AGCTACCTAACAGGTTTTGTTATAAAG	122
61	IV	E32M50	S: TCACATTAGTAAAACGATTGTCCAC<br>A: GATTGATTTTTTGGACTCCGTT	252
62	IV	OPN20	S: CCTTAGTTTAGTTGTAGGTGGTGG<br>A: AGAAACCGCTCAATTTTAACATAA	587

Sort	Marker Group	Coded Marker	Primer Sequence（5'-3'）	Size in bp
63	IV	OPH15	S: CCTTGGCTATGTGCTTATGTATTT<br>A: TAAAACACAGAGACAATCGTGAGG	637
64	IV	IN6RS4	S: CATTGATACATGAATGCAAAGAAG<br>A: GATGAAAACATTTACAGACAATGC	236
65	IV	E33M58	S: CTGCATAAAATTATCGAAGACAGATA<br>A: TTCTGTTTCAGCGCTAACAAATC	281
66	IV	E32M59A	S: CTTTGTCATTGTGTGTGTGTGTGT<br>A: AATATGATTTCCAATTTGCCAAGT	406
67	IV	E32M59B	S: AATTCTTGCTCCATTATGATTTCA<br>A: CACAAGACGATCAGGAAAAAGAA	350
68	IV	OPH03	S: TCCACTCCTAGTTCACAATCTATTTT<br>A: TATACAAAATGTTGGAATACACAAGG	591
69	V	RME01	S: TCCATTGCAGAATTCACCTG<br>A: TGTTTTCTTCGTCATGTCGG	454
70	V	RME02	S: CTTGAGGGAAGGAGACGAGA<br>A: ATTTTGGGTCATGGGTTTTT	233
71	V	RME03	S: ATATCCTTAAACCCTTGCGC<br>A: TTGAATACCTCCAAGGACCC	533
72	V	RME04	S: GGTCTCAGGTTTTGTGGGAG<br>A: GGTTCTCAAAGATTCCGAGG	699
73	V	RME05	S: CTTGGTCACACCCATCTTCTC<br>A: TGTCCGATAAACTCTCTGCG	477
74	V	RME06	S: ATCAACCACGTTCATCCATG<br>A: AACTCAAATACTCTCGGCCAG	480
75	V	RME07	S: ATTTACCAAATGGATCACTCTGG<br>A: CCGAGAATTGAACATTGTAAAGA	579
76	V	RME08	S: CAATTCCACAACGTAGCAGAG<br>A: CTTTTCGACTAAGAACCGGC	496
77	V	RME09	S: AGCTTGGACTATGCCGTTTG<br>A: ATTTCAGGACCGGCTATGTG	574
78	V	RME10	S: TCGAGAATCCTCTACAAACGC<br>A: AAGCACCACTTATTCGACAGC	570
79	V	IN10RS4	S: CAGAACACAGTTCTATGACACTG<br>A: TATAGGAGCTTTGTTCTGTAGTGG	287

Supplementary Table S2: Primer sequences for *Rfo* (*PPRB*) genes

Name	Primer Sequence（5'-3'）
PPRB-1	S: GTCCCAGATACTATCACCTA
	A: GAAGCTCTGCTGCGCAAACA
PPRB-2	S: TGACATGCTTCGATCTCGTC
	A: GAAGCTCTTGCTACCCATCG
PPRB-3	S: GCTCTGGTAGATGGAGCGAC
	A: TGGGACTATACCCCTGTGGG

Supplementary Table S3: Primer sequences for qRT-PCR.

Name	Primer Sequence（5'-3'）
ACTIN	S: CTATCCTCCGYCTCGATCTCGC
	A: CTTAGCCGTCTCCAGCTCTTGC
PPRB	S: TTGCGGATAGTAATGGTA
	A: GAGGGATAGTTGCTGATG

Supplementary Figure S1

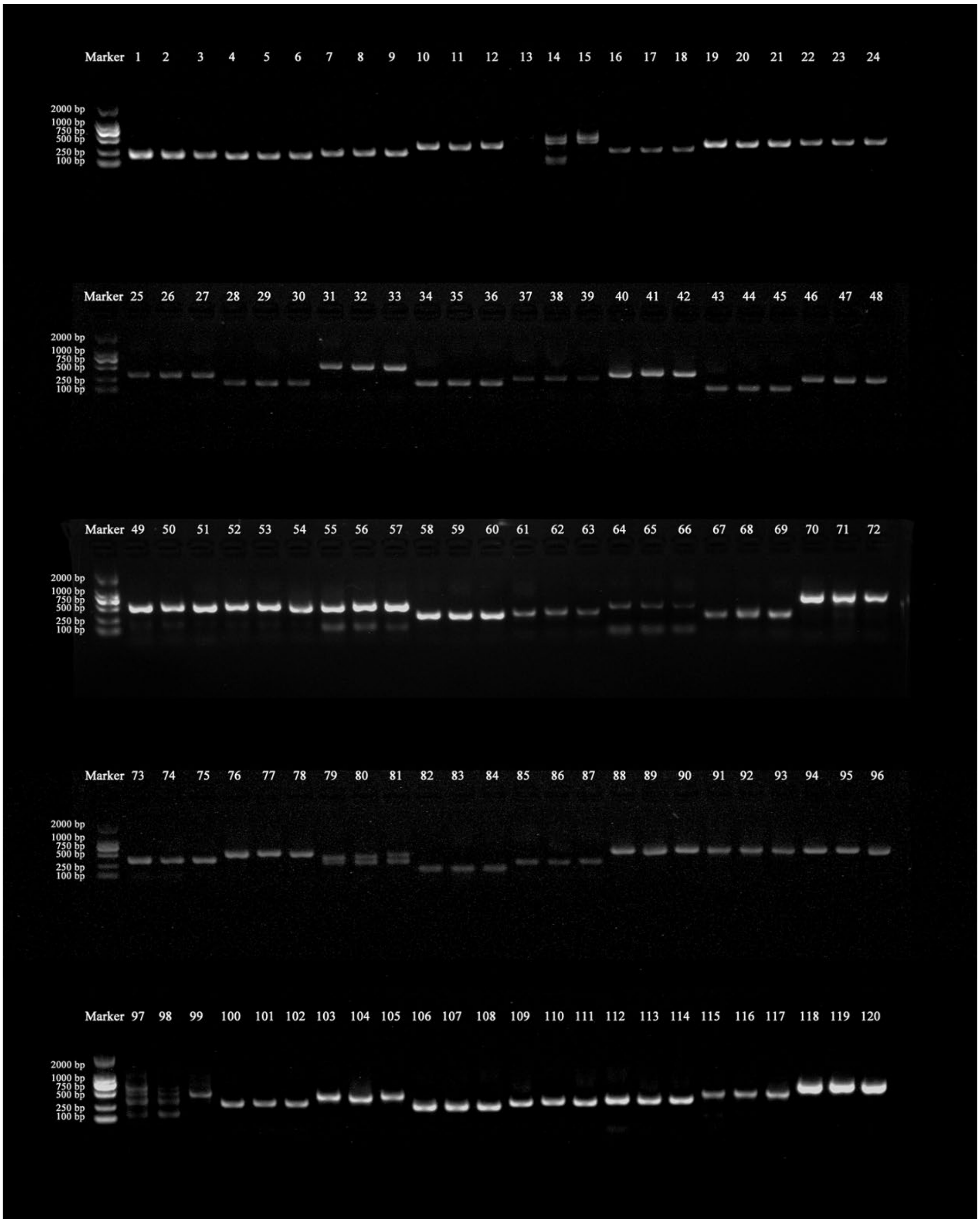

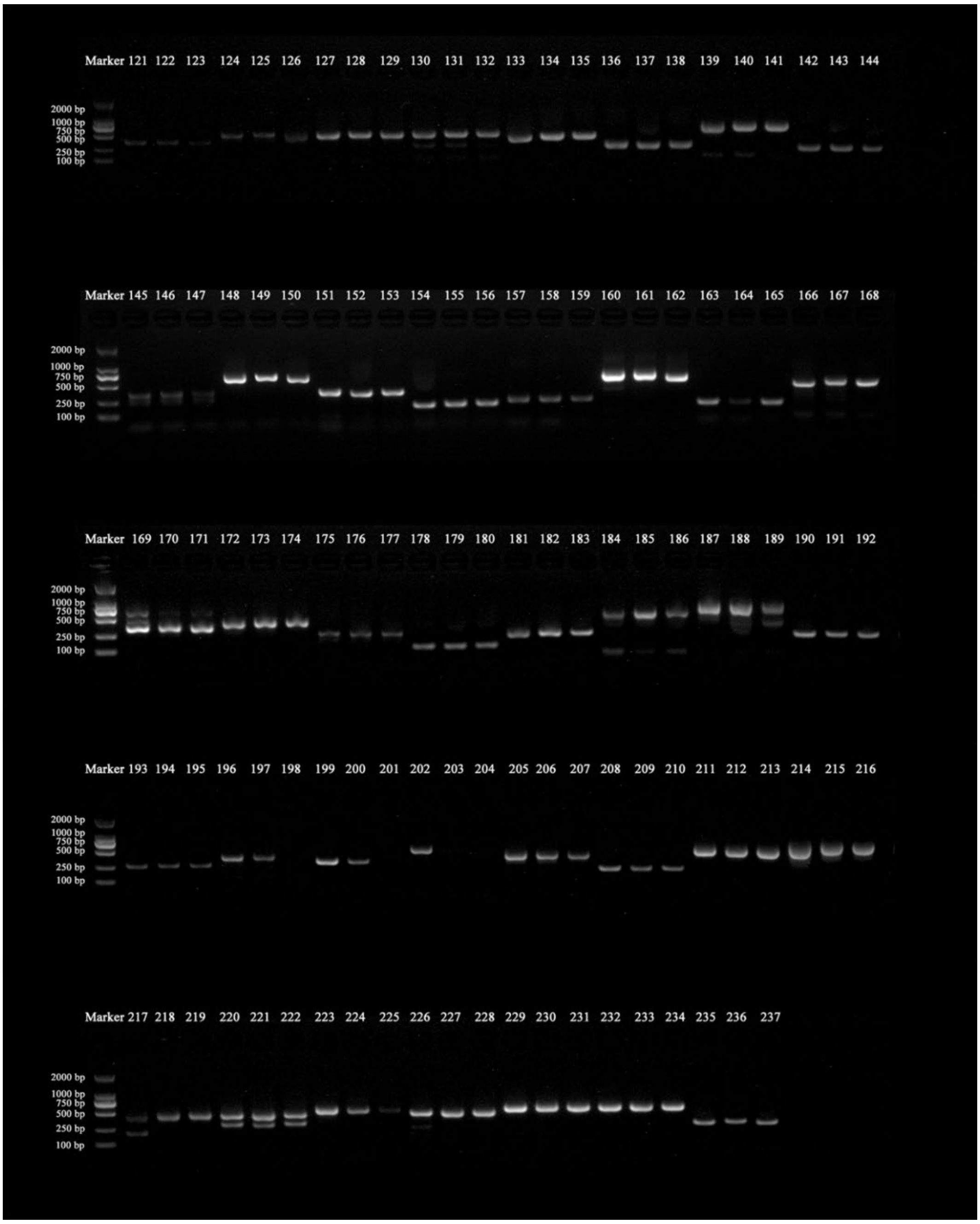

Figure S1 The Amplification results of 79 markers (listed in Supplementary Table 1) on R2000, CLR650 and Zhehuhong. 1-3 corresponds to the amplification of molecular markers RMA01 on Zhehuhong, CLR650 and R2000. 4-6 corresponds to the amplification of molecular markers RMA02 on Zhehuhong, CLR650 and R2000. 7-9 corresponds to the amplification of molecular markers RMA03 on Zhehuhong, CLR650 and R2000, and so on
